# The Impact of Healthcare-Associated Infection on Mortality: Failure in Clinical Recognition Is Related with Inadequate Antibiotic Therapy

**DOI:** 10.1371/journal.pone.0058418

**Published:** 2013-03-08

**Authors:** Teresa Cardoso, Orquídea Ribeiro, Irene Aragão, Altamiro Costa-Pereira, António Sarmento

**Affiliations:** 1 Intensive Care Unit – Unidade de Cuidados Intensivos Polivalente – Hospital Geral de Santo António, Abel Salazar Biomedical Sciences Institute, University of Porto, Porto, Portugal; 2 Department of Health Information and Decision Sciences, Center for Research in Health Technologies and Information Systems (CINTESIS), Faculty of Medicine, University of Porto, Porto, Portugal; 3 Intensive Care Unit – Unidade de Cuidados Intensivos Polivalente - Hospital Geral de Santo António, University of Porto, Porto, Portugal; 4 Department of Infectious Diseases, Hospital de São João, University of Porto, Porto, Portugal; Fundacion Huesped, Argentina

## Abstract

**Purpose:**

To understand if clinicians can tell apart patients with healthcare-associated infections (HCAI) from those with community-acquired infections (CAI) and to determine the impact of HCAI in the adequacy of initial antibiotic therapy and hospital mortality.

**Methods:**

One-year prospective cohort study including all consecutive infected patients admitted to a large university tertiary care hospital.

**Results:**

A total of 1035 patients were included in this study. There were 718 patients admitted from the community: 225 (31%) with HCAI and 493 (69%) with CAI. Total microbiologic documentation rate of infection was 68% (n = 703): 56% in CAI, 73% in HCAI and 83% in hospital-acquired infections (HAI). Antibiotic therapy was inadequate in 27% of patients with HCAI *vs.* 14% of patients with CAI (p<0.001). Among patients with HCAI, 47% received antibiotic therapy in accordance with international recommendations for treatment of CAI. Antibiotic therapy was inadequate in 36% of patients with HCAI whose treatment followed international recommendations for CAI *vs*. 19% in the group of HCAI patients whose treatment did not follow these guidelines (p = 0.014). Variables independently associated with inadequate antibiotic therapy were: decreased functional capacity (adjusted OR = 2.24), HCAI (adjusted OR = 2.09) and HAI (adjusted OR = 2.24). Variables independently associated with higher hospital mortality were: age (adjusted OR = 1.05, per year), severe sepsis (adjusted OR = 1.92), septic shock (adjusted OR = 8.13) and inadequate antibiotic therapy (adjusted OR = 1.99).

**Conclusions:**

HCAI was associated with an increased rate of inadequate antibiotic therapy but not with a significant increase in hospital mortality. Clinicians need to be aware of healthcare-associated infections among the group of infected patients arriving from the community since the existing guidelines regarding antibiotic therapy do not apply to this group and they will otherwise receive inadequate antibiotic therapy which will have a negative impact on hospital outcome.

## Introduction

According to the 2012 World Health Organization report, infections are among the top three leading causes of death worldwide [1], with community-acquired infections playing a major role.

At the end of the XX^th^ century, there was a significant increase in the healthcare service provided in the outpatient setting. As a consequence, the debate about the need to add a third category named “healthcare-associated infections” [2]–[4] to the existing dichotomy classification of community- and hospital-acquired infections, arose.

The classification of healthcare-associated infection proposed by Deborah Friedman in 2002 [2], is one of the most widely used in clinical studies. According to her, it is an infection present at the time of hospital admission or within 48 hours of admission, in patients who fulfill any of the following criteria:

Received intravenous therapy at home, wound care or specialized nursing care through a healthcare agency, family or friends; or, self-administered intravenous medical therapy in the 30 day period before the onset of the infection. Patients whose only home therapy was oxygen use are excluded.
Attended a hospital or haemodialysis clinic, or having received intravenous chemotherapy in the previous 30 days.
Were hospitalized in an acute care hospital for 2 or more days in the previous 90 days.
Resided in a nursing home or long-term care facility.

Since then, several studies have shown that the microbiologic profiles of healthcare-associated infections differ from those of community-acquired infections [2]–[4], and that this needs to be taken into account when prescribing first intention antibiotic therapy.If healthcare-associated infection are not recognized as a separate group among infected patients from the community, it might lead to inadequate first intention antibiotic therapy and worsen the prognosis.

The objectives of this study are: to understand if doctors differentiate between patients with healthcare-associated infections and those with community-acquired infections and to determine the impact of healthcare-associated infection in the adequacy of initial antibiotic therapy and hospital mortality.

## Methods

### Ethics Statement

This study was approved by the Institutional Review Board of Hospital de Santo António, Oporto Hospital Centre, Portugal, and informed consent was waived due to the observational nature of the study.

### Study Design and Patient Population

A prospective cohort study was conducted in a 600-bed tertiary care university hospital over the period of 1 year (1^st^ June 2008 to 31^th^ May 2009). All adult infected patients who were admitted to the medical, surgical, nephrology or hematology wards of the hospital or to the intensive care unit (ICU), whose infection fulfilled the CDC criteria [5], were included. Infections were classified as community-acquired infections (CAI), healthcare-associated infections (HCAI) or hospital-acquired (HAI), according to the place of acquisition.

### Definitions

**Community-acquired infections** **(CAI)** were defined as infections detected within 48 hours of hospital admission in patients who did not fit the criteria for a HCAI.

**Healthcare-associated infections (HCAI)** were defined by the same criteria that Deborah Friedman used for HCAI bloodstream infections [2], regardless of the involved focus of infection. This choice was based on the fact that this definition is widely used in similar studies and is not limited to bloodstream infections, but can also be applied to respiratory and specific pathogene infections [6]–[11].

**Hospital-acquired infections** **(HAI)** were defined as a localized or systemic condition that resulted from an adverse reaction to the presence of an infectious agent(s) or its toxin(s), that occurred within 48 hours or more after hospital admission and that was not incubating at the time of admission [12]. Infections in patients discharged from the hospital within the previous 2-week period were also included in this group.

The comorbidities of patients in the study included immunosuppression (administration of chemotherapy in the 12 months prior to hospital admission, either radiation therapy or administration of 0.2 mg/kg/day prednisolone for at least 3 months prior to hospital admission, administration of 1 mg/kg/day of prednisolone for 1 week in the 3 months prior to hospital admission or infection with human immunodeficiency virus), chronic liver diseases [13], chronic heart failure [13], chronic respiratory diseases [13], haematological diseases [14], cancer [14], diabetes mellitus requiring insulin therapy or oral hypoglycaemic agents before the infection and/or atherosclerosis (defined as a previous history of a transient ischemic attack, stroke, angina, myocardial infarction or peripheral arterial disease).

The general medical condition was assessed by the Karnofsky index [15]. A score of less than 70 implies that the patient is unable to perform normal activities or do active work.

“Hospitalization in the previous year” excluded patients already included in the group of patients with HCAI; that is, those with hospital admissions in the previous 3 months.

Sepsis and sepsis-related conditions were diagnosed according to the criteria proposed by the ACCP/SCCM [16].

The initial empirical antibiotic treatment was considered “adequate” if the antibiotic prescribed within the first 24 hours matched in vitro susceptibility of a pathogen deemed to be the likely cause of infection and when the dosage and route of administration were appropriate for current medical status (focus and severity of infection); only patients in which the pathogen was microbiologically identified were considered in this analysis.

To evaluate if doctors treated healthcare-associated infections as community-acquired infections, accordance of initial antibiotic therapy to the Infectious Diseases Society of America (IDSA) international recommendations for the treatment of community-acquired respiratory, urinary and intra-abdominal infections was assessed [17]–[19].

### Statistical Analysis

Continuous variables are described as means and standard deviations (SD). Categorical variables are described with absolute frequencies and percentages. Student *T*-tests or Mann-Whitney tests were used to compare continuous values. For categorical variables these comparisons were performed using Pearson χ^2^ test.

Variables associated with inadequate antibiotic therapy and hospital mortality were studied using logistic regression. Those with a clear association in the univariate analysis (*p*-value <0.1) were selected for the multivariable analysis. The results of the multivariable models are expressed as odds ratio (OR) with 95% confidence interval (CI_95%_) and p-values. The calibration was tested using the Hosmer-Lemeshow goodness-of-fit test. The significance level was defined as p<0.05.

Data was analyzed using SPSS, version 18 for Windows (Chicago, IL).

## Results

During the study period, a total of 3733 patients were admitted to the selected wards (103 beds); 1035 (28%) met the inclusion criteria of infections according to the CDC definitions of infection.

Of all patients included, 493 (48%) were diagnosed with CAI, 225 (22%) with HCAI and 317 (30%) with HAI.

Patients with HCAI were older, with a higher prevalence of previous comorbidities and inability to perform normal activities or do active work (Karnofsky index <70) than patients in the other two groups (p<0.05). They also had higher rate of admissions to hospital in the period between 3 and 12 months prior to the current episode, as well as previous antibiotic administration and inadequate antibiotic therapy, when compared to patients with CAI (p<0.001) (Table 1).

**Table 1:** Demographic and clinical characteristics of patients; comparing patients with healthcare-associated infections with patients with community or hospital-acquired infections.

Patients’ characteristics	TOTAL	HCAI	CAI	HAI	HCAI vs CAI	HCAI vs HAI
	(n = 1035)	(n = 225)	(n = 493)	(n = 317)	*p value*	*p value*
Age, mean (SD)	65 (20)	68 (19)	64 (20)	64 (19)	0.015^#^	0.022^#^
Male sex, n (%)	506 (49)	108 (48)	236 (48)	162 (51)	0.974*	0.476*
Severity of infection, n (%)					0.117*	0.243*
Infection	281 (27)	69 (31)	126 (26)	86 (27)		
Sepsis	364 (35)	73 (32)	178 (36)	113 (36)		
Severe sepsis	296 (29)	70 (31)	139 (28)	87 (27)		
Septic shock	94 (9)	13 (6)	50 (10)	31 (10)		
Previous comorbidities, n (%)	671 (65)	190 (84)	270 (55)	211 (67)	<0.001*	<0.001*
Karnofsky index<70, (%)	319 (31)	112 (50)	115 (23)	106 (33)	<0.001*	<0.001*
Hospitalization in the previous year (excluding the last 3 months, n (%)	413 (40)	55 (43)	91 (19)	174 (55)	<0.001*	0.027*
Previous antibiotic therapy, n (%)	367 (36)	86 (38)	51 (10)	230 (73)	<0.001*	<0.001*
Inadequate antibiotic therapy, n (%)	148 (21)	45 (27)	37 (14)	66 (25)	<0.001*	0.601*
Hospital mortality, n (%)	138 (13)	32 (14)	47 (10)	59 (19)	0.063*	0.178*

CAI – community-acquired infection, HCAI – healthcare-associated infection, HAI – hospital acquired infection, SD – Standard deviation. *Pearson Qui-square Test; # T-student test.

Total rate of microbiological documentation of infection was 68% (n = 703): 73% (n = 165) among patients with HCAI, 56% (n = 274) among those with CAI and 83% (n = 264) in the group with HAI.

The empirical antibiotic therapy was changed in 92 patients (13%) among those with microbiological documentation of infection (n = 703). The main reason for changing antibiotic therapy was adjustment to microbiology findings: 81% (n = 26) in HCAI *vs* 83% (n = 20) in CAI *vs* 89% (n = 32) in HAI. Other reasons for changing antibiotic therapy were a lack of clinical response in 11% (n = 10), side effects in 3% (n = 3) and others in 1% (n = 1).

In HCAI, 27% had received inadequate antibiotic therapy *vs.* 14% in CAI (p<0.001). Among patients with microbiologic documented HCAI, 47% (77) received antibiotic therapy according to international recommendations for CAI. Antibiotic therapy was inadequate in 36% of HCAI patients whose treatment followed the international recommendations for CAI *vs*. 19% in the group of HCAI patients whose treatment did not follow CAI treatment guidelines (p = 0.014).

The rate of inadequate antibiotic therapy among the four categories of HCAI was:

33% in the group of patients that received intravenous therapy at home, wound care or specialized nursing care through a healthcare agency, family or friends; or, self-administered intravenous medical therapy in the 30 day period before the onset of the infection;
21% in the group of patients that attended a hospital or hemodialysis clinic, or received intravenous chemotherapy in the previous 30 days*;*
30% in the group that were hospitalized in an acute care hospital for 2 or more days in the previous 90 days and
36% in those that resided in a nursing home or long-term care facility.

Variables significantly and independently associated with inadequate antibiotic therapy were Karnofsky index<70 [adjusted OR = 2.24 (CI_95%_ = 1.47–4.41)], HCAI [adjusted OR = 2.09 (CI_95%_ = 1.15–3.80)] and HAI [adjusted OR = 2.24 (CI_95%_ = 1.41–3.55)] (Tables 2 and 3).

**Table 2:** Variables associated with inadequate antibiotic therapy using logistic regression.

Variables	Total	Inadequate antibiotic therapy	Crude OR	CI_95%_	p- value	
	n = 703	n = 148				
Age, mean (SD)	65 (19)	69 (17)	1.02*	1.001–1.03-	0.063	
Sex, n (%)						
Female	363 (52)	75 (51)	1.00			
Male	340 (48)	73 (49)	1.05	0.73–1.51	0.793	
Previous antibiotic therapy, n (%)						
No	414 (59)	74 (50)	1.00			
Yes	289 (41)	74 (50)	1.58	1.10–2.28	0.014	
Hospitalization in the previous year (excluding the last 3 months), n (%)						
No	389 (62)	64 (50)	1.00			
Yes	241 (38)	64 (50)	1.83	1.24–2.72	0.001	
Comorbidities, n (%)						
No	222 (32)	38 (26)	1.00			
Yes	481 (68)	110 (74)	1.44	0.95–2.16	0.083	
Immunosupression, n (%)						
No	529 (75)	115 (78)	1.00			
Yes	174 (25)	33 (22)	0.84	0.55–1.30	0.437	
Chronic hepatic disease, n (%)						
No	686 (98)	143 (97)	1.00			
Yes	17 (2)	5 (3)	1.58	0.55–4.56	0.396	
Chronic heart failure, n (%)						
No	653 (93)	134 (90)	1.00			
Yes	50 (7)	14 (10)	1.51	0.79–2.87	0.214	
Chronic respiratory disease, n (%)						
No	661 (94)	41 (95)	1.00			
Yes	42 (6)	7 (5)	0.748	0.32–1.70	0.474	
Chronic haematologic disease, n (%)						
No	652 (93)	135 (91)	1.00			
Yes	51 (7)	13 (9)	1.31	0.70–2.53	0.421	
Cancer, n (%)						
No	670 (95)	136 (92)	1.00			
Yes	33 (5)	12 (8)	2.24	1.08–4.67	0.031	

* Increase in OR per year; SD – standard deviation, OR – *Odds ratio;* CI_95%_ - 95% confidence interval.

**Table 3:** Variables associated with inadequate antibiotic therapy using logistic regression.

Variables	Total	Inadequate antibiotic therapy	Crude OR	CI_95%_	p- value
	n = 703	n = 148			
Diabetes, n (%)					
No	558 (79)	119 (80)	1.00		
Yes	145 (21)	29 (20)	0.92	0.59–1.45	0.727
Atherosclerosis, n (%)					
No	539 (77)	101 (68)	1.00		
Yes	162 (23)	47 (32)	1.74	1.17–2.60	0.007
Karnovsky index<70, n (%)					
No	479 (68)	80 (54)	1.00		
Yes	224 (32)	68 (46)	2.17	1.50–3.16	<0.001
Type of infection					
Community-acquired, n (%)	274 (39)	37 (25)	1.00		
Healthcare-associated, n (%)	165 (23)	45 (30)	2.40	1.48–3.91	<0.001
Hospital-acquired, n (%)	264 (38)	66 (45)	2.14	1.37–3.33	0.001
Focus of infection					
Respiratory, n (%)	215 (31)	44 (30)	1.00		
Urinary, n (%)	306 (43)	69 (47)	1.13	0.74–1.73	0.570
Intra-abdominal, n (%)	124 (18)	24 (169)	0.93	0.55–1.63	0.806
Other, n (%)	58 (8)	11 (7)	0.91	0.44–1.90	0.801
Severity of infection					
Infection, n (%)	191 (27)	41 (28)	1.00		
Sepsis, n (%)	238 (34)	53 (36)	1.05	0.66–1.66	0.842
Severe sepsis, n (%)	209 (30)	43 (29)	0.95	0.59–1.53	0.827
Septic shock, n (%)	65 (9)	11 (7)	0.75	0.36–1.55	0.433

OR – *Odds ratio;* CI_95%_ - 95% confidence interval.

Variables significantly and independently associated with higher hospital mortality were age [adjusted OR = 1.05 (CI_95%_ = 1.03–1.07), per year], severe sepsis [adjusted OR = 1.92 (CI_95%_ = 1.01–3.63)], septic shock [adjusted OR = 8.13 (CI_95%_ = 3.84–17.23)] and inadequate antibiotic therapy [adjusted OR = 1.99 (CI_95%_ = 1.20–3.30)] (Table 4).

**Table 4:** Variables associated with hospital mortality using logistic regression.

Variables	Hospital mortality	Crude OR	CI_95%_	p- value	Adjusted OR	CI_95%_	
Age, mean (SD)	76 (14)	1.04*	1.03–1.06	<0.001	1.05*	1.03–1.07	
Sex, n (%)				0.036			
Female	59 (43)	1.00					
Male	79 (57)	1.47	1.03–2.12				
Previously healthy, n (%)				<0.001			
Yes	30 (22)	1.00					
No	108 (78)	2.14	1.39–3.27				
Type of infection							
Community-acquired, n (%)	47 (34)	1.00					
Healthcare-associated, n (%)	32 (23)	1.57	0.97–2.54	0.064			
Hospital-acquired, n (%)	59 (43)	2.17	1.44–3.28	<0.001			
Focus of infection							
Respiratory, n (%)	63 (4615)	1.00					
Urinary, n (%)	35 (2510)	0.64	0.41–0.99	0.047			
Intra-abdominal, n (%)	29 (2114)	0.89	0.55–1.43	0.632			
Other, n (%)	11 (819)	0.18	0.64–2.63	<0.001			
Severity of infection							
Infection, n (%)	20 (157)	1.00					
Sepsis, n (%)	30 (228)	1.17	0.65–2.11	0.597	0.980	0.50–1.92	
Severe sepsis, n (%)	46 (3316)	2.40	1.38–4.17	0.002	1.919	1.014–3.632	
Septic shock, n (%)	42 (3045)	10.54	5.73–19.40	<0.001	8.133	3.839–17.231	
Inadequate antibiotic therapy,n (%)	32 (32)	2.01	1.26–3.21	0.003	1.991	1.204–3.295	

* Increase in OR per year; OR – *Odds ratio*, CI_95%_ - 95% confidence interval.

The Hosmer and Lemeshow test did not show evidence of lack of fit in both models (p>0.1).

## Discussion

The results of this study show that doctors treat a high proportion (47%) of patients with healthcare-associated infections according to community-acquired infections treatment recommendations, implying that they do not differentiate this sub-group of patients among those with community-acquired infections.

Patients with healthcare-associated infections who were treated according to the international recommendations for community-acquired infections had a higher rate of inadequate antibiotic therapy than those in whom CAI treatment recommendations were not followed. Healthcare-associated infections were independently associated with inadequate antibiotic therapy. Inadequate antibiotic therapy was an independent risk factor for increased hospital mortality.

Decision making regarding antibiotic treatment is unique, no treatment equals its efficacy. In the meta-analysis on the efficacy of adequate antibiotic therapy for sepsis, by Paul et al [20], the pooled OR for all-cause mortality was 1.6, corresponding to a number needed to treat (NNT) of 10 to save one life, higher than aspirin in acute myocardial infarction (NNT = 41), reinforcing the need to get initial antibiotic therapy adequate in severe infection.

The adequate treatment of healthcare-associated infections is primarily dependent on the correct classification in patients that come from the community as CAI or HCAI. Previous studies [2]–[4] have shown that patients with HCAI have different microbiological profiles than those with CAI, namely a higher rate of multi-drug resistant pathogens [21], suggesting that the existing guidelines for CAI might not be applicable. Clinicians need to be aware of this difference in order to adapt empiric antibiotic therapy for HCAI patients.

There are a large number of epidemiological studies on healthcare-associated infections [3], [4], [6], [7], [9], [21]–[23] but the existing evidence needs to be systematized, in order to make information more profitable. Respiratory infections are the ones most widely addressed and the American Thoracic Society has already published specific recommendations for healthcare-associated pneumonia [24]; nevertheless, the wide-spectrum antibiotic therapy proposed for these patients has turned into a much debated subject where there is no consensus among clinicians. Additional studies are needed in this area, with thorough microbiologic characterization from different settings, before specific recommendations regarding empirical treatment for different focus of HCAI can be made.

HCAI was not found to be an independent risk factor for hospital mortality although the association of HCAI and higher mortality was described in the studies by Kollef et al [3] and Shorr et al [4]. However, none of them perform a multiple logistic regression to allow the statement of HCAI as an independent risk factor, as it was done in the present study.

There are several studies describing the negative impact of initial inadequate antibiotic therapy on mortality [25]–[32]. The results of the present study are in accordance with previous findings, from different centres and geographical areas, increasing the probability of wide external applicability of the main results.

Major strengths of this study are its prospective design with thorough data collection, the use of clear definitions in the protocol, data collection by a single trained doctor and full completion of all protocols with no missing data per item minimizing any information bias. Additionally, all patients completed the follow-up until hospital discharge. The similarity of some results with previous studies suggests an increased external application of the conclusions.

There are also some limitations that should be pointed out. The research was performed in a single institution and the number of patients with HCAI was relatively low. Only 68% of the patients had microbiologic documentation of infection and were evaluated for the adequacy of empirical antibiotic therapy; this may limit the logistic regression analysis in terms of its ability to detect all independent variables associated with inadequate antibiotic therapy and outcome. Nevertheless, the variables found were clinically significant and relevant, easily recognizable, and could function as additional aids to avoid inadequate therapy.

### Conclusions

HCAI were associated with an increased rate of inadequate antibiotic therapy, but not with a significant increase in hospital mortality.

Clinicians need to be aware of healthcare-associated infections among the group of infected patients arriving from the community, as the existing guidelines regarding antibiotic therapy do not apply to them. This is essential in order to prevent the associated inadequate antibiotic therapy and its negative impact on hospital outcome.

The development of specific guidelines for this group of infected patients should be considered.
